# Patella Alta on X-Ray and MRI: Diagnostic Agreement and Clinical Correlations

**DOI:** 10.3390/diagnostics16152444

**Published:** 2026-08-03

**Authors:** Faten Abdulrahman Almohideb, Mehad Hussni Felemban, Nizar Al-Nakshabandi, Mamdouh Saad Almalki, Mohammad Alfaqih, Abdullah Alfaqih

**Affiliations:** 1 Department of Radiology and Medical Imaging, College of Medicine, King Saud University Medical City, King Saud University, Riyadh 11472, Saudi Arabia; 2 Radiology Department, King Faisal Specialist Hospital & Research Centre, Riyadh 11211, Saudi Arabia

**Keywords:** patella alta, Insall–Salvati ratio, Patellotrochlear Index, magnetic resonance imaging, radiography

## Abstract

**Background/Objectives:** Patella alta contributes to patellofemoral (PF) maltracking and anterior knee pain, but the agreement of patellar-height indices across imaging modalities and their clinical relevance remain uncertain. We aimed to estimate the prevalence of patella alta when using each index, quantify inter-method agreement, and quantify patella alta’s associations with Hoffa’s fat-pad impingement, patellar chondromalacia, and tibial tubercle–trochlear groove (TT–TG) distance. **Methods:** This retrospective cross-sectional study included 250 patients aged 18–38 years who underwent both knee radiography and MRI. Patellar height was assessed using the Insall–Salvati (IS) and modified Insall–Salvati (mIS) ratios on radiographs and MRI, and the Patellotrochlear Index (PTI) on MRI. Associations with fat-pad impingement, chondromalacia grade, and TT–TG abnormality were analyzed using the chi-square or Fisher’s exact test and as odds ratios (ORs) adjusted for age, sex, and imaged side; agreement was assessed with Cohen’s kappa (κ). **Results:** Patella alta prevalence was 13.2% using IS on radiographs and 10.8% using IS on MRI, but markedly lower with mIS (≤1.6%). Patella alta was more frequent in patients with Hoffa’s fat-pad impingement (31.1% vs. 5.7% on IS radiography; adjusted OR 8.83, 95% CI 3.82–20.45) and advanced chondromalacia (*p* = 0.025). Agreement was moderate between IS radiography and IS MRI (κ = 0.47, 95% CI 0.33–0.61) and between IS MRI and the PTI (κ = 0.43). **Conclusions:** The prevalence and clinical relevance of patella alta depend strongly on the measurement method. The IS ratio was the most consistent index, but radiography–MRI agreement was moderate rather than high, so the two are not interchangeable. The PTI adds information on trochlear engagement from the same MRI examination. These cross-sectional associations do not establish causation.

## 1. Introduction

The patellofemoral (PF) joint is frequently affected by structural disorders that contribute significantly to anterior knee pain and the development of patellofemoral osteoarthritis (PFOA) [1]. Among the main anatomical risk factors for PF instability and adverse outcomes are trochlear dysplasia, abnormal patellar height, and excessive tibial tubercle–trochlear groove (TT–TG) distance [2,3,4].

Patella alta, defined as a high-riding patella relative to the trochlear groove, is a critical component of PF pathology [5]. A highly positioned patella can decrease the contact area between the patellar articular surface and the trochlea, particularly during knee extension, potentially increasing shear forces and contributing to patellar instability and PFOA risk [6].

Patella alta is also implicated in other localized pathologies, notably Hoffa’s fat-pad impingement, where the high position of the patella is associated with microtrauma and edema in the superolateral portion of the fat pad. Additionally, patella alta is present in approximately 25–30% of patients experiencing acute patellar dislocations [7].

Accurate measurement of patellar height is crucial for clinical evaluation and surgical planning, especially when procedures such as tibial tubercle distalization are considered [8]. However, there is no current consensus on the optimal measurement method or imaging modality. Several indices exist, including the original Insall–Salvati (IS) ratio, the modified Insall–Salvati (mIS) ratio, the Caton–Deschamps Index (CDI), and the Blackburne–Peel Index (BPI) [7]. These methods were primarily developed for conventional radiography (XR). With the increasing use of magnetic resonance imaging (MRI) in assessing knee structural damage, indices like the IS ratio are now commonly applied to MRI measurements [9].

The Patellotrochlear Index (PTI), specifically designed for MRI, offers a direct measurement of patellar–trochlear cartilage overlap, reflecting functional engagement. The utility of applying traditional radiographic ratios (IS, mIS) to cross-sectional modalities like MRI, and establishing their comparability, remains an important clinical and methodological consideration.

Furthermore, patellar height rarely acts in isolation. Patellofemoral stability is defined by constraints provided by soft tissues and chondral/bony geometry [10]. Malalignment, quantified primarily by the TT–TG distance, is a concurrent risk factor, measuring the lateralization of the tibial tuberosity [11]. Understanding the co-occurrence and combined effects of patella alta and TT–TG abnormalities is essential for a complete multi-parameter patellofemoral assessment.

Given these complexities, this retrospective study of young adults who underwent both knee radiography and MRI had three prespecified objectives: (i) to estimate the prevalence of patella alta defined separately by the IS and mIS ratios on radiographs and on MRI, and by the PTI on MRI; (ii) to quantify inter-method agreement between the radiographic and MRI indices, and between the MRI height ratios and the PTI, using Cohen’s kappa with 95% confidence intervals; and (iii) to quantify the strength of association between patella alta and Hoffa’s fat-pad impingement, patellar chondromalacia grade, and TT–TG distance, before and after adjustment for age, sex, and imaged side.

## 2. Materials and Methods

### 2.1. Study Design and Setting

We conducted a retrospective cross-sectional imaging study at King Khalid University Hospital (King Saud University Medical City, Riyadh, Saudi Arabia), reviewing the radiology archive and electronic medical records from 1 January 2018 to 31 December 2021. The institutional review board approved the study with a waiver of informed consent owing to its retrospective, minimal-risk design (approval No. E-25-10311). All data were de-identified prior to analysis and handled in accordance with institutional and national data-protection policies.

### 2.2. Participants

Consecutive patients with both knee MRI and lateral knee radiography performed within the study window were screened. Eligible participants were 18–38 years old and had technically adequate imaging acquired at the institution. Patients were excluded if they had undergone prior knee surgery, or if they had acute traumatic injury at presentation, inflammatory arthropathy, congenital or developmental anomalies affecting patellofemoral morphology (such as bipartite patella), neoplasm, or poor-quality/incomplete imaging. When both knees were imaged, one knee was randomly selected a priori to preserve independence. All patients followed the same clinical imaging pathway and shared a single indication for imaging: chronic, persistent, unexplained knee pain in the absence of any history of acute trauma. Lateral knee radiography was obtained first as the baseline examination, and knee MRI was requested subsequently by the treating clinician when symptoms persisted and remained unexplained. Patients presenting with acute traumatic injury were excluded, as stated above, so the cohort contains no acute-injury subgroup. No examination was performed for research purposes, and no patient was imaged solely to measure patellar height.

### 2.3. Imaging Acquisition

**Figure 1:**
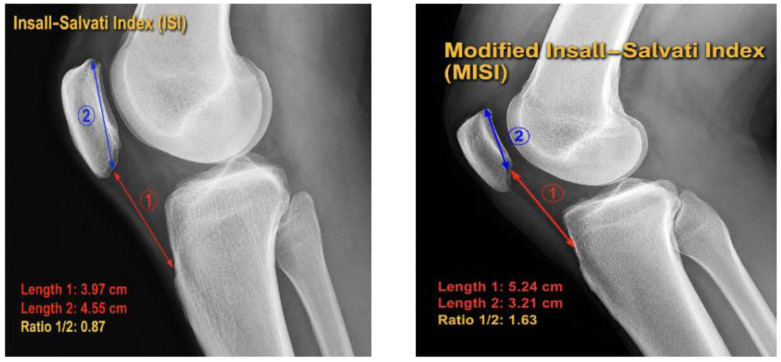
X-ray assessment of patellar height.

**Figure 2:**
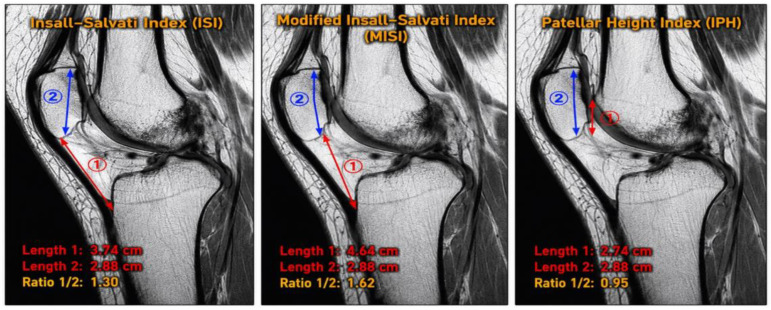
MRI assessment of patellar height and engagement.

Standard anteroposterior (AP) and true lateral knee radiographs were obtained per protocol, with lateral images acquired at ~20–30° flexion when feasible. MRI was performed on 1.5-T or 3-T scanners (SIGNA Architect, GE HealthCare, Chicago, IL, USA) using routine patellofemoral sequences, including sagittal T1-weighted and fat-suppressed fluid-sensitive images in the sagittal, axial, and coronal planes. Available technical parameters (field strength, slice thickness, and in-plane resolution) and the radiograph–MRI time interval were recorded. Patellar height indices were measured digitally on the picture archiving and communication system (PACS) using standardized calipers and magnification, as illustrated in Figure 1 (radiographic assessment) and Figure 2 (MRI assessment). In every patient the MRI examination followed the radiograph within an interval of four to six weeks.

On radiographs, the Insall–Salvati (IS) ratio was calculated as patellar tendon length divided by patellar length (patella alta > 1.2), and the modified Insall–Salvati (mIS) ratio as tendon attachment-to-articular surface distance divided by articular surface length (patella alta > 2.0). On MRI, the same indices were measured with higher thresholds (IS > 1.4, mIS > 2.4). The Patellotrochlear Index (PTI) was calculated as the trochlear cartilage overlap length divided by total patellar cartilage length, with patella alta defined as PTI < 0.18. Patella baja was defined as IS < 0.8 on radiographs and IS < 0.74 on MRI, and as mIS < 1.24 on MRI; values between the baja and alta thresholds were classified as normal. Modality-specific thresholds were used because a patellar-height ratio measured on a single sagittal MRI slice is not interchangeable with the projectional measurement obtained on a lateral radiograph: slice selection, degree of knee flexion, and the conspicuity of the tendon insertion all shift the ratio upward on MRI, so that the classical radiographic cut-off of 1.2 over-diagnoses patella alta when transferred directly to MRI. Shabshin et al. derived MRI-specific reference values in 245 patients and reported cut-offs of >1.50 for alta and <0.74 for baja on MRI, compared with the radiographic values originally defined by Insall and Salvati [12]. That study also reported sex-specific MRI alta thresholds of 1.32 in men and 1.52 in women. Because the present cohort was predominantly male (69.6%) and included both sexes, an intermediate MRI threshold of 1.4 was prespecified for the primary analysis, lying between the male and female cut-offs reported by Shabshin et al.; the baja threshold of 0.74 was taken directly from that study. The prevalence and agreement analyses were repeated using the more conservative overall threshold of 1.50 as a sensitivity analysis (Section 3.7).

### 2.4. Measurements and Operational Definitions

Patellar height served as the exposure. The IS ratio and the mIS ratio were measured on the lateral radiograph and, independently, on sagittal MRI for every patient; no radiographic value was substituted from MRI, and all 250 patients contributed a complete set of radiographic and MRI measurements. The PTI was measured on sagittal MRI. Patella alta was defined dichotomously using thresholds prespecified in the analysis plan; secondary analyses modeled each index as a continuous predictor and applied alternative published cut-points. Outcomes were defined on MRI. Patellar chondromalacia was graded on fat-suppressed fluid-sensitive MRI using the modified Outerbridge classification [13]: grade 0, normal cartilage; grade 1, intact cartilage surface with focal signal hyperintensity indicating softening or swelling; grade 2, superficial fraying, blistering, or focal defects involving less than 50% of cartilage thickness; grade 3, partial-thickness cartilage loss with ulceration, fissuring, or fragmentation involving more than 50% of cartilage thickness without subchondral marrow edema; and grade 4, full-thickness cartilage loss with exposed subchondral bone and reactive marrow edema or subchondral cysts. For analysis, grade 0 was classified as normal, grades 1–2 as early chondromalacia, and grades 3–4 as advanced chondromalacia. Hoffa’s fat-pad impingement was recorded based on edema-like signal and characteristic impingement features and analyzed as present versus absent. Patellar maltracking was assessed by the TT–TG distance measured on axial MRI and classified using prespecified cut-offs as normal (<15 mm), borderline (15–20 mm), or abnormal (>20 mm), with the 20 mm value being the threshold originally identified as pathological by Dejour et al. [14]; the continuous TT–TG value was retained for additional models. Covariates available for all patients, and therefore carried into the adjusted models, were age, sex, and imaged side. The radiograph–MRI interval was four to six weeks in every patient and was therefore effectively constant across the cohort, so it could not act as a confounder and was not entered as a covariate. The indication for imaging was likewise uniform by design (Section 2.2).

### 2.5. Reader Workflow and Quality Control

All measurements were performed by a single fellowship-trained musculoskeletal radiologist with more than five years of post-fellowship experience, who was blinded to the clinical records at the time of measurement. Because each examination was assessed by one reader, interobserver reliability could not be calculated; this is acknowledged as a limitation.

### 2.6. Outcomes

The primary outcome was the prevalence of patella alta as determined by radiographic and MRI indices. Secondary outcomes included the associations between patella alta and each of Hoffa’s fat-pad impingement, patellar maltracking (TT–TG distance), and the grade of patellar chondromalacia, as well as the diagnostic agreement between X-ray and MRI measurements and with the PTI.

### 2.7. Statistical Analysis

Data entry and statistical analysis were conducted using the Statistical Package for the Social Sciences (SPSS), version 28 (IBM Corp., Armonk, NY, USA). Qualitative variables were summarized as frequencies and percentages, while quantitative variables were presented as range, mean, and standard deviation (SD). The chi-square test was applied to assess associations between categorical variables; because several contingency tables contained expected cell counts below five, Fisher’s exact test was computed for every such table and both *p*-values are reported. The magnitude of each association was quantified as an odds ratio (OR) with a 95% confidence interval (CI) obtained from binary logistic regression, with patella alta (versus not alta) as the exposure and fat-pad impingement, chondromalacia, and abnormal TT–TG distance as outcomes; models were fitted unadjusted and then adjusted for age, sex, and imaged side. Cohen’s kappa (κ) was used to evaluate inter-method agreement and is reported with its 95% CI and with overall percentage agreement, interpreted following Landis and Koch as slight (≤0.20), fair (0.21–0.40), moderate (0.41–0.60), substantial (0.61–0.80), or almost perfect (0.81–1.00) [15]. Because a significant kappa *p*-value shows only that agreement exceeds chance, and says nothing about its magnitude, agreement is described throughout by the value of κ rather than by its *p*-value. A prespecified sensitivity analysis repeated the MRI prevalence and agreement analyses using an IS threshold of 1.5 instead of 1.4. A *p*-value < 0.05 was considered statistically significant.

## 3. Results

The study included 250 patients aged 18–38 years (mean ± SD: 28.04 ± 5.70 years). Most patients were male (174, 69.6%), and more than half had right-sided involvement (54.4%) (Table 1). All 250 patients had complete radiographic and MRI patellar-height measurements. Fat-pad impingement status could not be determined in one patient, and the TT–TG distance was not measurable in one other patient; analyses involving either of these two variables are therefore based on 249 patients, which accounts for the differing denominators (*n* = 250 or *n* = 249) stated in the heading of each table.

### 3.1. Prevalence of Patella Alta

As shown in Table 2, the prevalence of patella alta was 13.2% on IS X-ray and 10.8% on IS MRI, whereas it was much lower using the mIS method (1.6% on X-ray and 1.2% on MRI).

### 3.2. Fat-Pad Impingement Syndrome

**Figure 3:**
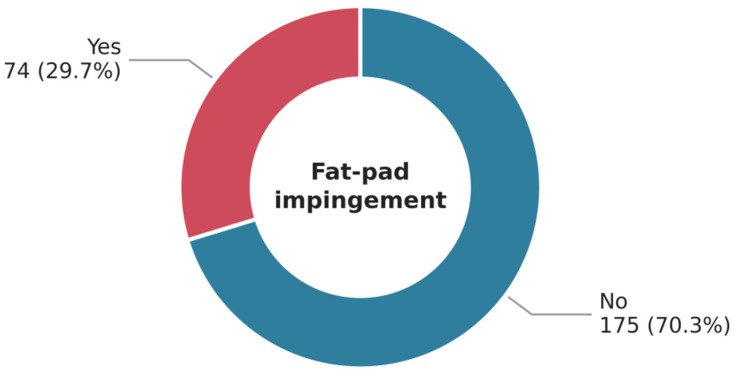
Prevalence of fat-pad impingement syndrome among patients who underwent both knee MRI and knee X-ray at King Khalid University Hospital (2018–2021) (*n* = 249).

As shown in Figure 3, fat-pad impingement syndrome was identified in 29.7% of patients who underwent both knee MRI and X-ray. Patella alta was significantly more frequent among patients with fat-pad impingement: 31.1% vs. 5.7% using IS X-ray (*p* < 0.001) and 23.0% vs. 5.7% using IS MRI (*p* < 0.001). Using mIS X-ray, patella alta was also more common in patients with fat-pad impingement (4.1% vs. 0.6%), although this comparison was significant only on the chi-square test (*p* = 0.046) and not on Fisher’s exact test (*p* = 0.080), which is the appropriate test given that one expected cell count was below five. With mIS MRI, the association was not statistically significant according to either test (Table 3). In terms of magnitude, patella alta on IS X-ray was associated with an approximately sevenfold higher odds of fat-pad impingement (crude OR 7.44, 95% CI 3.32–16.66; adjusted OR 8.83, 95% CI 3.82–20.45), patella alta on IS MRI with a fivefold higher odds (crude OR 4.92, 95% CI 2.13–11.37; adjusted OR 5.56, 95% CI 2.34–13.20), and patella alta on the PTI with a sevenfold higher odds (crude OR 7.25, 95% CI 2.48–21.17; adjusted OR 8.33, 95% CI 2.74–25.39) (Table 4).

### 3.3. Tibial Tubercle–Trochlear Groove Measurement

**Figure 4:**
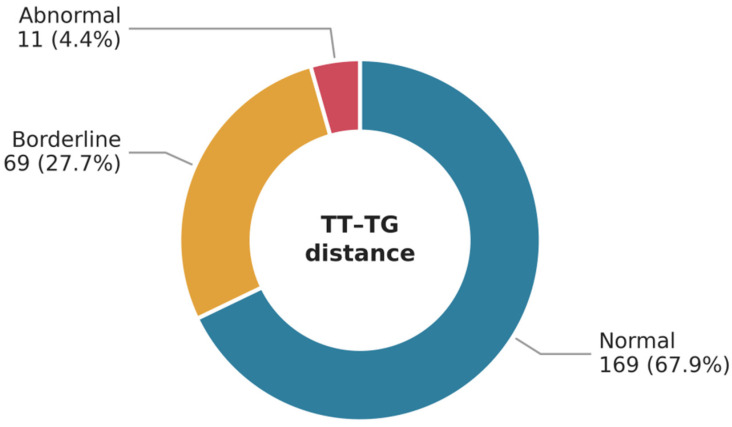
Tibial tubercle–trochlear groove measurement among patients who underwent both knee MRI and knee X-ray at King Khalid University Hospital (2018–2021) (*n* = 249).

The prevalence of borderline and abnormal TT–TG measurements was 27.7% and 4.4%, respectively (Figure 4). Patella alta was significantly more common among patients with abnormal TT–TG compared with those with normal TT–TG when assessed by IS X-ray (36.4% vs. 11.8%, *p* = 0.014) and by mIS MRI (9.1% vs. 0.0%, *p* = 0.003). In contrast, using IS MRI and mIS X-ray, patella alta was observed more frequently in patients with abnormal TT–TG, but the differences did not reach statistical significance on the chi-square test (Table 5). Expected cell counts were below five in every one of these comparisons, so Fisher’s exact test was also computed: the association with IS X-ray remained significant (*p* = 0.005), as did that with mIS MRI (*p* = 0.008), while the mIS X-ray comparison reached the conventional threshold (*p* = 0.049) and the IS MRI comparison did not (*p* = 0.071). Comparing abnormal TT–TG with normal or borderline TT–TG, patella alta on IS X-ray was associated with a fourfold higher odds of abnormal TT–TG (crude OR 4.12, 95% CI 1.14–14.94; adjusted OR 3.90, 95% CI 1.05–14.51), but with only 11 patients in the abnormal group this estimate is imprecise and should be regarded as hypothesis-generating (Table 4).

### 3.4. Agreement with the Patellotrochlear Index

**Figure 5:**
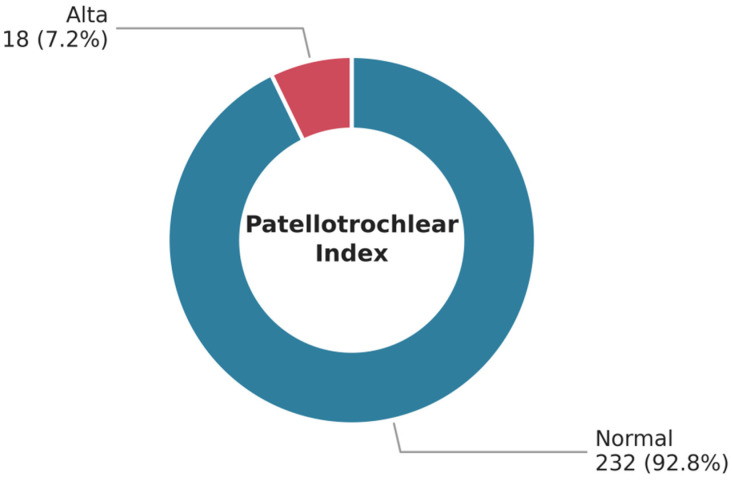
Patellotrochlear Index among patients who underwent both knee MRI and knee X-ray at King Khalid University Hospital (2018–2021) (*n* = 250).

According to the PTI, patella alta was observed in 7.2% of patients (Figure 5). Agreement between IS MRI and the PTI was statistically significant but moderate in magnitude (κ = 0.43, 95% CI 0.25–0.61; overall agreement 89.6%; *p* < 0.001): patella alta was diagnosed by both methods in only 44.4% of IS-MRI alta cases, although normal findings were confirmed in 97.2% of IS-MRI normal cases. Agreement between mIS MRI and the PTI was also statistically significant but only slight (κ = 0.14, 95% CI −0.05 to 0.34; overall agreement 92.0%; *p* = 0.001), with concordant patella alta in 66.7% of the three mIS-MRI alta cases and concordant normal findings in 93.4%. These figures illustrate that the apparently high raw concordance is driven almost entirely by the large number of jointly normal cases, and that the two MRI approaches classify patella alta itself quite differently (Table 6).

### 3.5. Grade of Chondromalacia

**Figure 6:**
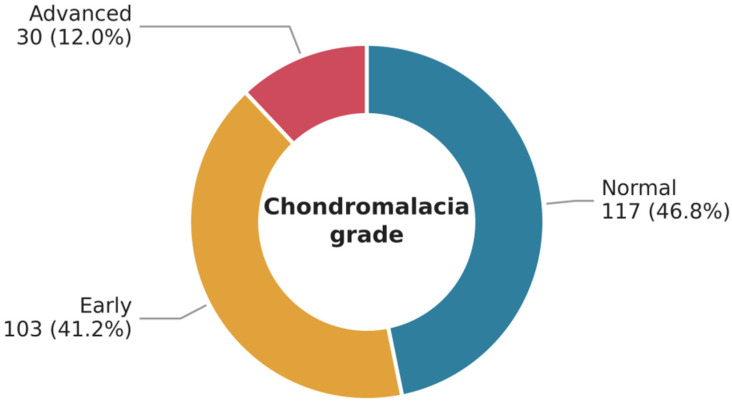
Grade of chondromalacia among patients who underwent both knee MRI and knee X-ray at King Khalid University Hospital (2018–2021) (*n* = 250).

Figure 6 shows that early chondromalacia was present in 41.2% of patients, while advanced chondromalacia was observed in 12.0%. Patella alta was significantly more frequent among patients with advanced chondromalacia compared with those with normal cartilage when assessed by IS X-ray (23.3% vs. 6.0%, *p* = 0.025; Fisher’s exact *p* = 0.019) and IS MRI (13.3% vs. 3.4%, *p* = 0.009; Fisher’s exact *p* = 0.002). In contrast, no significant association between patella alta and chondromalacia grade was detected using the mIS X-ray or MRI methods (Table S1). When chondromalacia of any grade was compared with normal cartilage, patella alta was associated with a higher odds of cartilage abnormality on both IS measurements (IS X-ray: crude OR 3.82, 95% CI 1.59–9.17, adjusted OR 4.74, 95% CI 1.91–11.75; IS MRI: crude OR 5.91, 95% CI 1.98–17.63, adjusted OR 8.76, 95% CI 2.79–27.49); for advanced chondromalacia specifically, the association reached significance only for the radiographic IS after adjustment (adjusted OR 3.15, 95% CI 1.14–8.70) (Table 4).

### 3.6. Agreement Between Insall–Salvati Measurements on X-Ray and MRI

Agreement between IS X-ray and IS MRI was statistically significant and moderate in magnitude (κ = 0.47, 95% CI 0.33–0.61; overall agreement 84.8%; *p* < 0.001), with concordant diagnoses in 54.5% of patella alta cases and 95.0% of normal cases. Agreement between mIS X-ray and mIS MRI was also moderate (κ = 0.59, 95% CI 0.23–0.96; overall agreement 98.4%; *p* < 0.001), with patella alta confirmed by both methods in 75.0% of cases and normal findings in 98.8% of cases; the very wide confidence interval reflects the fact that only four radiographic and three MRI cases were classified as alta by the mIS method, and this estimate should not be interpreted as evidence of strong cross-modality reliability (Table S2).

### 3.7. Sensitivity Analysis and Adjusted Associations

Repeating the MRI analyses with the more conservative threshold of IS > 1.5 reduced the MRI prevalence of patella alta from 10.8% to 4.0% (10 of 250 patients) and lowered agreement with the radiographic IS from moderate to fair (κ = 0.38, 95% CI 0.20–0.56), while the association with fat-pad impingement remained significant (9.5% vs. 1.7%, *p* = 0.004). The direction of every association was therefore unchanged by the choice of MRI threshold, but the estimated prevalence was more than halved, which reinforces the point that prevalence figures for patella alta are threshold-dependent and are not comparable across studies using different cut-offs. Table 4 presents the magnitude of each association as crude and adjusted odds ratios. After adjustment for age, sex, and imaged side, patella alta remained independently associated with fat-pad impingement for all three definitions and with chondromalacia of any grade for both IS measurements; for advanced chondromalacia the association reached significance only for the radiographic IS, and the estimates for abnormal TT–TG distance were compatible with a substantial effect but too imprecise to be conclusive.

## 4. Discussion

In this young adult cohort (mean age ≈ 28 years), patella alta prevalence varied widely by metric and modality: it was higher with IS on radiographs and MRI, but markedly lower with mIS. Patella alta was significantly over-represented in patients with fat-pad impingement and in those with advanced chondromalacia, particularly when assessed by IS (XR and MRI). TT–TG abnormalities were uncommon but, when present, co-occurred with more patella alta on IS-XR and mIS-MRI. Agreement between IS-MRI and the PTI, and between XR and MRI for IS, was moderate (κ = 0.43 and κ = 0.47, respectively) rather than high, indicating that the modalities give related but not interchangeable classifications; the apparently near-perfect concordance of the mIS ratio across modalities rests on only a handful of alta cases and carries a confidence interval too wide to support a claim of cross-modality consistency. These patterns align with contemporary imaging guidance that treats patellar height as one component of a multi-parameter patellofemoral assessment [16].

That IS identified substantially more alta than mIS is expected from how these ratios behave. IS is a tendon-to-bone ratio (patellar tendon length ÷ patellar length) and is comparatively robust across modalities; multiple comparative studies show IS is the only height metric with good intra-/inter-observer reliability on conventional radiography, CT, and MRI and the best XR–MRI agreement, while other ratios (including many mIS variants) suffer from poorer inter-method agreement due to landmarking and definitional variability [17]. Seil et al. state that the mIS method presents a disadvantage compared with the IS method, namely the inherent difficulty in identifying the distal end of the patellar articular surface [18]; recent reviews recommend acknowledging that tendon/bone and engagement metrics describe related but distinct constructs [16].

Hoffa’s fat pad, as an intra-capsular structure located inferior to the patella, can explain our finding that patella alta was markedly over-represented in Hoffa’s fat-pad impingement [19]. One proposed mechanism is that a high-riding patella applies traction on Hoffa’s fat pad, with secondary edema and fibrotic change. Because the present design is retrospective and cross-sectional, our data can show only that the two findings occur together; they cannot establish that patella alta produced the fat-pad change, and neither the reverse sequence nor a shared underlying cause can be excluded. Whether correcting patellar height alters the course of Hoffa’s fat-pad syndrome would need to be tested prospectively. A recent systematic review concluded that high patellar height and increased TT–TG predispose patients to Hoffa’s fat-pad syndrome, which is consistent with the association observed in our data [20]. Clinically, our results identify alta as a marker that merits attention in patients with fat-pad symptoms (e.g., targeted rehabilitation to improve engagement, taping/bracing strategies); when multimodal maltracking metrics cluster (alta ± elevated TT–TG), timely referral for PF-focused evaluation is reasonable [16,20].

Although overtly abnormal TT–TG was uncommon, its presence coincided with higher alta rates (significant with IS-XR and mIS-MRI). This co-clustering matches the multi-parameter model of instability: vertical malposition (alta) amplifies the lateralizing vector measured by TT–TG [21,22].

The graded relationship we observed—alta associated with advanced chondromalacia on IS-XR and IS-MRI—aligns with longitudinal and cross-sectional MRI analyses linking patella alta to worsening patellofemoral cartilage damage/OA progression over 24 months in population cohorts [23]. Additional MRI studies connect malalignment/maltracking to patellar cartilage injury and altered cartilage composition, consistent with a proposed biologic pathway in which a high-riding patella engages the femoral groove less effectively, with focal cartilage overload and progressive biochemical degeneration reflected as longer T1ρ/T2 times and worse KOOS outcomes [24]; that pathway is plausible but cannot be tested in a cross-sectional dataset such as ours. That mIS did not discriminate severity in our data reinforces the notion that tendon-bone ratios (IS) may better track the maltracking phenotype driving PF cartilage stress, whereas mIS variability can attenuate signal [17].

Our finding of significant agreement between the PTI and IS-MRI/mIS-MRI is noteworthy. Comparative studies have emphasized limited correlation between tendon/bone ratios and the PTI because they quantify different constructs [25,26,27]; however, when the MRI technique is standardized and the cohort is relatively homogeneous (as in our young adults), concordance can improve, as the data show.

The moderate XR–MRI agreement for IS in our study is directionally consistent with the largest reliability synthesis to date: IS is the most reliable height metric across CR/CT/MRI and the only one with consistently acceptable cross-modality agreement, while other indices show only poor-to-moderate inter-method concordance. One explanation may be that IS uses only bony references and therefore can be measured more consistently across different imaging modalities [23,24,25]. Practically, this supports using IS when radiographic and MRI datasets have to be compared, provided the modality-specific thresholds are applied and the two values are not treated as equivalent for an individual patient. It also clarifies what MRI adds over radiography for patellar height. The IS ratio is not intrinsically more accurate on MRI; its value on MRI is that it is obtained from the same examination that displays cartilage, fat pad, and TT–TG, and that it can be paired with the PTI, which measures articular engagement and cannot be derived from a radiograph at all. Our findings therefore do not imply that both examinations should be requested for the sole purpose of measuring patellar height.

### 4.1. Strengths and Limitations

This study has several limitations. Its retrospective, single-center design limits generalizability and causal inference, and selection bias is possible because only imaged patients were included. Imaging findings could not be confirmed surgically or arthroscopically. Minor variability in MRI field strength and radiographic flexion angle may have affected quantitative measurements. Residual confounding is possible due to unavailable clinical factors (e.g., BMI, activity level, limb alignment). Additionally, the small number of patients with abnormal TT–TG distance limited subgroup precision. Four further limitations should be noted. All measurements were made by a single reader, so interobserver reliability could not be quantified and measurement error cannot be estimated. Several comparisons, in particular those involving the mIS indices and the abnormal TT–TG group, rested on expected cell counts below five; these were re-analyzed with Fisher’s exact test, but the resulting estimates remain imprecise, as the width of the confidence intervals in Table 4 shows. All patients were imaged for the same indication, which limits indication bias but also means the findings apply specifically to young adults with chronic unexplained knee pain and should not be extrapolated to acute injury or to asymptomatic populations. Finally, the adjusted models included only age, sex, and imaged side, and residual confounding by unmeasured factors remains possible.

Despite this, the study has important strengths. It is among the few to directly compare IS, mIS, and the PTI across both MRI and radiography in the same cohort. Blinding of the reader to the clinical records reduced measurement bias, and predefined analyses with a sensitivity analysis at an alternative MRI threshold improved internal validity. The cohort was also clinically homogeneous: every patient was imaged for the same indication and underwent both examinations within a fixed four-to-six-week window, which reduces indication bias and makes the radiographic and MRI measurements directly comparable. Consistent results across modalities support the robustness of the findings.

### 4.2. Clinical Implications and Future Directions

The findings of this study support the Insall–Salvati (IS) index as the most practical of the indices examined for measuring patellar height on both radiography and MRI, showing stronger associations with fat-pad impingement, abnormal TT–TG distance, and advanced chondromalacia. In contrast, the modified IS (mIS) may underestimate clinically relevant patella alta and should be used cautiously when interpreted alone.

The observed agreement between IS and the MRI-based PTI supports reporting both when an MRI has already been performed, since they capture tendon-based patellar height and articular-based trochlear engagement respectively. Because the PTI is obtained from the same examination, this adds no additional imaging, cost, or radiation exposure, and our results should not be read as a recommendation to obtain both radiography and MRI in order to measure patellar height. Clinically, identifying patella alta may help guide tailored rehabilitation and, when needed, realignment strategies, although the present cross-sectional data cannot show that modifying patellar height changes those outcomes.

Future studies should confirm these results in prospective, multi-center cohorts with standardized imaging techniques and surgical correlation, and explore whether early correction of patellar height abnormalities can reduce progression of patellofemoral cartilage degeneration.

## 5. Conclusions

In young symptomatic adults, the measured prevalence of patella alta and the apparent strength of its clinical correlates depend strongly on which index and which modality are used. Patella alta defined by the IS ratio was associated with Hoffa’s fat-pad impingement (adjusted OR 8.83 on radiographs) and with patellar chondromalacia, and these associations persisted after adjustment for age, sex, and imaged side, whereas the mIS ratio identified too few cases to show any association. Because this study is retrospective and cross-sectional, these are associations only and do not establish that patella alta causes fat-pad or cartilage change. Agreement between the radiographic and MRI IS measurements was moderate (κ = 0.47) rather than high, so the two are not interchangeable and a given patient should be followed up with the same index on the same modality. The PTI agreed only moderately with IS-MRI (κ = 0.43) and measures a different construct, articular engagement. When an MRI has already been obtained for a clinical indication, reporting the IS ratio alongside the PTI adds information at no extra cost or radiation exposure; these findings do not support performing both radiography and MRI for the sole purpose of measuring patellar height.

## Figures and Tables

**Table 1:** Age, sex, and affected side of patients who underwent both knee MRI and knee X-ray at King Khalid University Hospital (2018–2021).

Characteristic	Value
Age (years)	
Range	18–38
Mean ± SD	28.04 ± 5.70
**Sex, *n* (%)**	
Male	174 (69.6)
Female	76 (30.4)
**Affected side, *n* (%)**	
Right	136 (54.4)
Left	114 (45.6)

SD, standard deviation.

**Table 2:** Prevalence of patella alta among patients who underwent both knee MRI and knee X-ray at King Khalid University Hospital (2018–2021) (*n* = 250).

	Frequency	Percentage
**Insall–Salvati (X-ray)**		
Baja	17	6.8
Normal	200	80.0
Alta	33	13.2
**Insall–Salvati (MRI)**		
Baja	5	2.0
Normal	218	87.2
Alta	27	10.8
**Modified Insall–Salvati (X-ray)**		
Baja	0	0.0
Normal	246	98.4
Alta	4	1.6
**Modified Insall–Salvati (MRI)**		
Baja	3	1.2
Normal	244	97.6
Alta	3	1.2

Baja, patella baja (infera).

**Table 3:** Association between patella alta and fat-pad impingement syndrome (*n* = 249).

**Insall–Salvati (X-ray)**				
	**Baja (N = 17), *n* (%)**	**Normal (N = 199), *n* (%)**	**Alta (N = 33), *n* (%)**	*p*-Value *
**No (*n* = 175)**	14 (8.0)	151 (86.3)	10 (5.7)	<0.001 (<0.001)
**Yes (*n* = 74)**	3 (4.1)	48 (64.9)	23 (31.1)	
**Insall–Salvati (MRI)**				
	**Baja (N = 5), *n* (%)**	**Normal (N = 217), *n* (%)**	**Alta (N = 27), *n* (%)**	*p*-Value *
**No (*n* = 175)**	5 (2.9)	160 (91.4)	10 (5.7)	<0.001 (<0.001)
**Yes (*n* = 74)**	0 (0.0)	57 (77.0)	17 (23.0)	
**Modified Insall–Salvati (X-ray)**				
	**Baja (N = 0), *n* (%)**	**Normal (N = 245), *n* (%)**	**Alta (N = 4), *n* (%)**	*p*-Value *
**No (*n* = 175)**	—	174 (99.4)	1 (0.6)	0.046 (0.080)
**Yes (*n* = 74)**	—	71 (95.9)	3 (4.1)	
**Modified Insall–Salvati (MRI)**				
	**Baja (N = 3), *n* (%)**	**Normal (N = 243), *n* (%)**	**Alta (N = 3), *n* (%)**	*p*-Value *
**No (*n* = 175)**	3 (1.7)	171 (97.7)	1 (0.6)	0.199 (0.219)
**Yes (*n* = 74)**	0 (0.0)	72 (97.3)	2 (2.7)	

* Chi-square test. Baja, patella baja (infera). Values in parentheses are *p*-values from Fisher’s exact test, reported because one or more expected cell counts were below five.

**Table 4:** Crude and adjusted associations between patella alta and knee MRI findings (binary logistic regression).

Exposure	Crude OR (95% CI)	Adjusted OR (95% CI) *	*p*-Value †
**Hoffa’s fat-pad impingement (*n* = 249)**			
Patella alta, IS X-ray	7.44 (3.32–16.66)	8.83 (3.82–20.45)	<0.001
Patella alta, IS MRI	4.92 (2.13–11.37)	5.56 (2.34–13.20)	<0.001
Patella alta, PTI	7.25 (2.48–21.17)	8.33 (2.74–25.39)	<0.001
**Chondromalacia of any grade (*n* = 250)**			
Patella alta, IS X-ray	3.82 (1.59–9.17)	4.74 (1.91–11.75)	<0.001
Patella alta, IS MRI	5.91 (1.98–17.63)	8.76 (2.79–27.49)	<0.001
**Advanced chondromalacia (*n* = 250)**			
Patella alta, IS X-ray	2.27 (0.89–5.81)	3.15 (1.14–8.70)	0.027
Patella alta, IS MRI	1.32 (0.42–4.11)	1.67 (0.50–5.58)	0.403
**Abnormal TT–TG distance (*n* = 249)**			
Patella alta, IS X-ray	4.12 (1.14–14.94)	3.90 (1.05–14.51)	0.043
Patella alta, IS MRI	3.34 (0.83–13.46)	3.36 (0.80–14.10)	0.097

* Adjusted for age, sex, and imaged side. † *p*-value for the adjusted odds ratio. For each exposure the reference category is absence of patella alta for that index. CI, confidence interval; IS, Insall–Salvati ratio; OR, odds ratio; PTI, Patellotrochlear Index; TT–TG, tibial tubercle–trochlear groove.

**Table 5:** Association between patella alta and tibial tubercle–trochlear groove measurement (*n* = 249).

**Insall–Salvati (X-ray)**				
	**Baja (N = 17), *n* (%)**	**Normal (N = 199), *n* (%)**	**Alta (N = 33), *n* (%)**	*p*-Value *
**Normal (*n* = 169)**	16 (9.5)	133 (78.7)	20 (11.8)	0.014 (0.005)
**Borderline (*n* = 69)**	0 (0.0)	60 (87.0)	9 (13.0)	
**Abnormal (*n* = 11)**	1 (9.1)	6 (54.5)	4 (36.4)	
**Insall–Salvati (MRI)**				
	**Baja (N = 5), *n* (%)**	**Normal (N = 217), *n* (%)**	**Alta (N = 27), *n* (%)**	*p*-Value *
**Normal (*n* = 169)**	4 (2.4)	149 (88.2)	16 (9.5)	0.090 (0.071)
**Borderline (*n* = 69)**	0 (0.0)	61 (88.4)	8 (11.6)	
**Abnormal (*n* = 11)**	1 (9.1)	7 (63.6)	3 (27.3)	
**Modified Insall–Salvati (X-ray)**				
	**Baja (N = 0), *n* (%)**	**Normal (N = 245), *n* (%)**	**Alta (N = 4), *n* (%)**	*p*-Value *
**Normal (*n* = 169)**	—	168 (99.4)	1 (0.6)	0.057 (0.049)
**Borderline (*n* = 69)**	—	67 (97.1)	2 (2.9)	
**Abnormal (*n* = 11)**	—	10 (90.9)	1 (9.1)	
**Modified Insall–Salvati (MRI)**				
	**Baja (N = 3), *n* (%)**	**Normal (N = 243), *n* (%)**	**Alta (N = 3), *n* (%)**	*p*-Value *
**Normal (*n* = 169)**	2 (1.2)	167 (98.8)	0 (0.0)	0.003 (0.008)
**Borderline (*n* = 69)**	0 (0.0)	67 (97.1)	2 (2.9)	
**Abnormal (*n* = 11)**	1 (9.1)	9 (81.8)	1 (9.1)	

* Chi-square test. Baja, patella baja (infera). Values in parentheses are *p*-values from Fisher’s exact test, reported because one or more expected cell counts were below five.

**Table 6:** Agreement between Insall–Salvati (MRI), modified Insall–Salvati (MRI), and the Patellotrochlear Index (*n* = 250).

**Insall–Salvati (MRI)**				
	**Baja (N = 5), *n* (%)**	**Normal (N = 218), *n* (%)**	**Alta (N = 27), *n* (%)**	*p*-Value *
**PTI Normal (*n* = 232)**	5 (100)	212 (97.2)	15 (55.6)	<0.001
**PTI Alta (*n* = 18)**	0 (0.0)	6 (2.8)	12 (44.4)	
**Modified Insall–Salvati (MRI)**				
	**Baja (N = 3), *n* (%)**	**Normal (N = 244), *n* (%)**	**Alta (N = 3), *n* (%)**	*p*-Value *
**PTI Normal (*n* = 232)**	3 (100)	228 (93.4)	1 (33.3)	0.001
**PTI Alta (*n* = 18)**	0 (0.0)	16 (6.6)	2 (66.7)	

* Cohen’s kappa test for agreement.

## Data Availability

The data presented in this study are available on request from the corresponding author. The data are not publicly available due to privacy and ethical restrictions.

## Supplementary Materials

The following supporting information can be downloaded at: https://www.mdpi.com/article/10.3390/diagnostics16152444/s1. Table S1: Association between patellar height category and chondromalacia grade among patients who underwent both knee MRI and knee X-ray at King Khalid University Hospital, 2018–2021 (*n* = 250); Table S2: Agreement between X-ray and MRI for the Insall–Salvati and modified Insall–Salvati indices in classifying patellar height (*n* = 250).

## Abbreviations

The following abbreviations are used in this manuscript: PF, patellofemoral; PFOA, patellofemoral osteoarthritis; MRI, magnetic resonance imaging; XR, radiography; IS ratio, Insall–Salvati ratio; mIS ratio, modified Insall–Salvati ratio; PTI, Patellotrochlear Index; TT–TG, tibial tubercle–trochlear groove; Baja, patella baja (infera); SD, standard deviation; CDI, Caton–Deschamps Index; BPI, Blackburne–Peel Index; OR, odds ratio; CI, confidence interval; κ, Cohen’s kappa.

## References

[1] Kobayashi S., Pappas E., Fransen M., Refshauge K., Simic M. (2016). The prevalence of patellofemoral osteoarthritis: A systematic review and meta-analysis. Osteoarthr. Cartil..

[2] Dietrich T.J., Fucentese S.F., Pfirrmann C.W.A. (2016). Imaging of individual anatomical risk factors for patellar instability. Semin. Musculoskelet. Radiol..

[3] Diederichs G., Issever A.S., Scheffler S. (2010). MR imaging of patellar instability: Injury patterns and assessment of risk factors. RadioGraphics.

[4] Berruto M., Ferrua P., Carimati G., Uboldi F., Gala L. (2013). Patellofemoral instability: Classification and imaging. Joints.

[5] Simmons E., Cameron J.C. (1992). Patella alta and recurrent dislocation of the patella. Clin. Orthop. Relat. Res..

[6] Ward S.R., Terk M.R., Powers C.M. (2007). Patella alta: Association with patellofemoral alignment and changes in contact area during weight-bearing. J. Bone Jt. Surg. Am..

[7] Biedert R.M., Tscholl P.M. (2017). Patella alta: A comprehensive review of current knowledge. Am. J. Orthop..

[8] Clark D., Walmsley K., Schranz P., Mandalia V. (2017). Tibial tuberosity transfer in combination with medial patellofemoral ligament reconstruction: Surgical technique. Arthrosc. Tech..

[9] Yue R.A., Arendt E.A., Tompkins M.A. (2017). Patellar height measurements on radiograph and magnetic resonance imaging in patellar instability and control patients. J. Knee Surg..

[10] Post W.R., Fithian D.C. (2018). Patellofemoral instability: A consensus statement from the AOSSM/PFF Patellofemoral Instability Workshop. Orthop. J. Sports Med..

[11] Nukala V., Sodhi A., Wadhavkar I., Mangudi Varadarajan K., Muratoglu O., Borjali A., Tanaka M.J. (2025). A magnetic resonance imaging–based clinical prediction model accurately identifies patellar instability risk using common patellofemoral measurements. Arthrosc. Sports Med. Rehabil..

[12] Shabshin N., Schweitzer M.E., Morrison W.B., Parker L. (2004). MRI criteria for patella alta and baja. Skelet. Radiol..

[13] Slattery C., Kweon C.Y. (2018). Classifications in brief: Outerbridge classification of chondral lesions. Clin. Orthop. Relat. Res..

[14] Dejour H., Walch G., Nove-Josserand L., Guier C. (1994). Factors of patellar instability: An anatomic radiographic study. Knee Surg. Sports Traumatol. Arthrosc..

[15] Landis J.R., Koch G.G. (1977). The measurement of observer agreement for categorical data. Biometrics.

[16] Barbosa R.M., Da Silva M.V., Macedo C.S., Santos C.P. (2023). Imaging evaluation of patellofemoral joint instability: A review. Knee Surg. Relat. Res..

[17] Verhulst F.V., Van Sambeeck J.D.P., Olthuis G.S., Van Der Ree J., Koëter S. (2020). Patellar height measurements: Insall–Salvati ratio is most reliable method. Knee Surg. Sports Traumatol. Arthrosc..

[18] Seil R., Müller B., Georg T., Kohn D., Rupp S. (2000). Reliability and interobserver variability in radiological patellar height ratios. Knee Surg. Sports Traumatol. Arthrosc..

[19] Gallagher J., Tierney P., Murray P., O’Brien M. (2005). The infrapatellar fat pad: Anatomy and clinical correlations. Knee Surg. Sports Traumatol. Arthrosc..

[20] Abelleyra Lastoria D.A., Benny C.K., Hing C.B. (2023). Predisposing factors for Hoffa’s fat pad syndrome: A systematic review. Knee Surg. Relat. Res..

[21] Friedman M.V., Hillen T.J., Misra S., Hildebolt C.F., Rubin D.A. (2020). Quantitative variable assessment of patellar instability: An MRI-based study. AJR Am. J. Roentgenol..

[22] Vairo G.L., Moya-Angeler J., Siorta M.A., Anderson A.H., Sherbondy P.S. (2019). Tibial tubercle–trochlear groove distance is a reliable and accurate indicator of patellofemoral instability. Clin. Orthop. Relat. Res..

[23] Haj-Mirzaian A., Guermazi A., Pishgar F., Pourvaziri A., Roemer F.W., Sereni C., Hakky M., Zikria B., Stefanik J., Demehri S. (2019). Association of patella alta with worsening of patellofemoral osteoarthritis-related structural damage: Data from the Osteoarthritis Initiative. Osteoarthr. Cartil..

[24] Liao T.C., Pedoia V., Link T.M., Majumdar S., Souza R.B. (2023). Association of patella alignment with cartilage relaxation times and self-reported symptoms in individuals with patellofemoral degeneration. J. Orthop. Res..

[25] Snow M., Singh N., Rix L., Haikal M. (2024). No correlation exists between tibial- and femoral-based measurements of patella alta in a population with chronic patellofemoral pain or instability undergoing patella distalization. Arthroscopy.

[26] Barnett A.J., Prentice M., Mandalia V., Wakeley C.J., Eldridge J.D.J. (2009). Patellar height measurement in trochlear dysplasia. Knee Surg. Sports Traumatol. Arthrosc..

[27] Ali S.A., Helmer R., Terk M.R. (2009). Patella alta: Lack of correlation between patellotrochlear cartilage congruence and commonly used patellar height ratios. AJR Am. J. Roentgenol..

