# Supplementary material for: Patella Alta on X-Ray and MRI: Diagnostic Agreement and Clinical Correlations
Source: Diagnostics (Basel). 2026 Aug 3;16(15):2444. doi: 10.3390/diagnostics16152444 (PMC13465340; doi:10.3390/diagnostics16152444)
Supplement: Supplementary file 1 [file diagnostics-16-02444-s001.zip › diagnostics-4422655-supplementary.pdf]

Supplementary Materials

Patella Alta on X-ray and MRI: Diagnostic Agreement and Clinical Correlations

Table S1. Association between patellar height category and chondromalacia grade among patients who underwent both knee MRI and knee X-ray at King Khalid University Hospital, 2018–2021 (n = 250).

**Insall–Salvati (X-ray)**				
	**Baja (n = 17), n (%)**	**Normal (n = 200), n (%)**	**Alta (n = 33), n (%)**	**p-value ***
**Normal (n = 117)**	10 (8.5)	100 (85.5)	7 (6.0)	0.025 (0.019)
**Early (n = 103)**	6 (5.8)	78 (75.7)	19 (18.4)	
**Advanced (n = 30)**	1 (3.3)	22 (73.3)	7 (23.3)	
**Insall–Salvati (MRI)**				
	**Baja (n = 5), n (%)**	**Normal (n = 218), n (%)**	**Alta (n = 27), n (%)**	**p-value ***
**Normal (n = 117)**	2 (1.7)	111 (94.9)	4 (3.4)	0.009 (0.002)
**Early (n = 103)**	2 (1.9)	82 (79.6)	19 (18.4)	
**Advanced (n = 30)**	1 (3.3)	25 (83.3)	4 (13.3)	
**Modified Insall–Salvati (X-ray)**				
	**Baja (n = 0), n (%)**	**Normal (n = 246), n (%)**	**Alta (n = 4), n (%)**	**p-value ***
**Normal (n = 117)**	—	117 (100.0)	0 (0.0)	0.165 (0.134)
**Early (n = 103)**	—	100 (97.1)	3 (2.9)	
**Advanced (n = 30)**	—	29 (96.7)	1 (3.3)	
**Modified Insall–Salvati (MRI)**				
	**Baja (n = 3), n (%)**	**Normal (n = 244), n (%)**	**Alta (n = 3), n (%)**	**p-value ***
**Normal (n = 117)**	2 (1.7)	115 (98.3)	0 (0.0)	0.289 (0.391)
**Early (n = 103)**	1 (1.0)	99 (96.1)	3 (2.9)	
**Advanced (n = 30)**	0 (0.0)	30 (100.0)	0 (0.0)	

* Chi-square test. Values in parentheses in the p-value column are p-values from Fisher's exact test, reported because one or more expected cell counts were below five. Percentages are row percentages, calculated within each chondromalacia grade. In the modified Insall–Salvati (X-ray) block no patient was classified as baja, so the reported p-value refers to a 3 × 2 table. Baja, patella baja (infera); n, number of patients.

Table S2. Agreement between X-ray and MRI for the Insall–Salvati and modified Insall–Salvati indices in classifying patellar height (n = 250).

**Insall–Salvati (X-ray)**				
	**Baja (n = 17), n (%)**	**Normal (n = 200), n (%)**	**Alta (n = 33), n (%)**	**p-value; κ (95% CI) ***
**IS-MRI Baja (n = 5)**	4 (23.5)	1 (0.5)	0 (0.0)	< 0.001; κ = 0.47 (0.33–0.61)
**IS-MRI Normal (n** = 218**)**	13 (76.5)	190 (95.0)	15 (45.5)	
**IS-MRI Alta (n** = 27**)**	0 (0.0)	9 (4.5)	18 (54.5)	
**Modified Insall–Salvati (X-ray)**				
	**Baja (n = 0), n (%)**	**Normal (n = 246), n (%)**	**Alta (n = 4), n (%)**	**p-value; κ (95% CI) ***
**mIS-MRI Baja (n = 3)**	—	3 (1.2)	0 (0.0)	< 0.001; κ = 0.59 (0.23–0.96)

**mIS-MRI Normal (n = 244)**	—	243 (98.8)	1 (25.0)	
**mIS-MRI Alta (n = 3)**	—	0 (0.0)	3 (75.0)	

* Cohen’s kappa test for agreement. Cohen’s kappa (κ) with its 95% confidence interval is reported alongside the p-value, since the p-value indicates only that agreement exceeds chance and does not describe its magnitude. Percentages are column percentages, calculated within each X-ray category. In the modified Insall–Salvati (X-ray) index no patient was classified as baja. CI, confidence interval; IS, Insall–Salvati; mIS, modified Insall–Salvati; n, number of patients.
